# Nutlin-3 overcomes arsenic trioxide resistance and tumor metastasis mediated by mutant p53 in Hepatocellular Carcinoma

**DOI:** 10.1186/1476-4598-13-133

**Published:** 2014-05-31

**Authors:** Tongsen Zheng, Dalong Yin, Zhaoyang Lu, Jiabei Wang, Yuejin Li, Xi Chen, Yingjian Liang, Xuan Song, Shuyi Qi, Boshi Sun, Changming Xie, Xianzhi Meng, Shangha Pan, Jiaren Liu, Hongchi Jiang, Lianxin Liu

**Affiliations:** 1 Key Laboratory of Hepatosplenic Surgery, Ministry of Education, Department of General Surgery, the First Affiliated Hospital of Harbin Medical University, #23 Youzheng Street, Harbin 150001, Heilongjiang Province, China; 2 Department of Gerontology, the First Affiliated Hospital of Harbin Medical University, Harbin, China; 3 Children’s Hospital Boston and Harvard Medical School, Boston, 300 Longwood Ave, Boston, MA 02115-5737, USA

**Keywords:** Arsenic trioxide resistance, Nutlin-3, Metastasis, p53 mutation, p73, Hepatocellular carcinoma

## Abstract

**Background:**

Arsenic trioxide has been demonstrated as an effective anti-cancer drug against leukemia and solid tumors both *in vitro* and *in vivo*. However, recent phase II trials demonstrated that single agent arsenic trioxide was poorly effective against hepatocellular carcinoma (HCC), which might be due to drug resistance.

**Methods:**

Mutation detection of p53 gene in arsenic trioxide resistant HCC cell lines was performed. The therapeutic effects of arsenic trioxide and Nutlin-3 on HCC were evaluated both *in vitro* and *in vivo*. A series of experiments including MTT, apoptosis assays, co-Immunoprecipitation, siRNA transfection, lentiviral infection, cell migration, invasion, and epithelial-mesenchy-mal transition (EMT) assays were performed to investigate the underlying mechanisms.

**Results:**

The acquisition of p53 mutation contributed to arsenic trioxide resistance and enhanced metastatic potential of HCC cells. Mutant p53 (Mutp53) silence could re-sensitize HCC resistant cells to arsenic trioxide and inhibit the metastatic activities, while mutp53 overexpression showed the opposite effects. Neither arsenic trioxide nor Nutlin-3 could exhibit obvious effects against arsenic trioxide resistant HCC cells, while combination of them showed significant effects. Nutlin-3 can not only increase the intracellular arsenicals through inhibition of p-gp but also promote the p73 activation and mutp53 degradation mediated by arsenic trioxide. *In vivo* experiments indicated that Nutlin-3 can potentiate the antitumor activities of arsenic trioxide in an orthotopic hepatic tumor model and inhibit the metastasis to lung.

**Conclusions:**

Acquisitions of p53 mutations contributed to the resistance of HCC to arsenic trioxide. Nutlin-3 could overcome arsenic trioxide resistance and inhibit tumor metastasis through p73 activation and promoting mutant p53 degradation mediated by arsenic trioxide.

## Background

Arsenic trioxide is a documented environmental toxicant and a potent chemotherapeutic agent, which has been used therapeutically for decades [1]. Besides being an anticancer drug against acute promyelocytic leukemia (APL), arsenic trioxide has also been proved as an effective compound that can inhibit the growth of many solid tumors both *in vitro* and *in vivo*[1,2]. It has been reported that liver is the most important site of arsenic biotransformation by alternating reduction of pentavalent arsenic to trivalent and addition of a methyl group from *S*-adenosylmethionine [3]. This evidence suggests that the hepatocellular carcinoma (HCC) cells could be more likely to be killed as a result of the aggregation of intracellular arsenicals after arsenic trioxide treatment. However, a recent phase II trial showed that single agent arsenic trioxide was poorly effective against advanced liver cancer with failure to increase the five-year survival rates [4], which was inconsistent with the *in vitro* reports. Although lots of factors could explain the inefficacy of arsenic trioxide in liver cancer patients, anti-cancer drug resistance might be the most important reason for this problem [5].

The increased drug efflux is defined as a characteristic of the multidrug resistant phenotype. Overexpression of transporters from ATP-binding cassette (ABC) superfamily is one of the most common reasons contributed to drug resistance. It was reported that the As-GSH conjugates is substrates of some ABC transporter proteins and could be pumped out by the ABC superfamily members [6,7]. In our previous study, we found that arsenic trioxide resistant HCC cells overexpressed p-glycoprotein (p-gp), which could decrease the intra-cellular arsenicals [5]. The resistant cells also overexpressed MDM2, which could inactivate p53 or p73, leading to the defence of apoptosis induced by arsenic trioxide. Interestingly, the expression of p53 was increased in arsenic trioxide resistant cells, suggesting there might be p53 mutations, which could lead to the stabilization of p53. In the current study, we hypothesized that long-term exposure of cells to arsenic trioxide in the stepwise selection of arsenic trioxide resistant HCC cells induced p53 mutations, which can result in arsenic trioxide resistance. Fortunately, unlike p53, another member of the p53 family, p73, is rarely mutated in cancers [8]. In addition, a few stimuli, including arsenic trioxide, have been identified to induce p73 and subsequent apoptosis in cancer cells [8,9]. However, although arsenic trioxide could induce p73, some negative moderators of p73, such as mutant p53 (mutp53) and MDM2 can suppress the apoptotic function of p73 [10]. As reported, the most common p53 mutation is single amino acid substitutions in the DNA binding domain of the p53 protein. In addition to the loss of tumor suppressive activities of wild-type p53, many tumor-associated mutp53 proteins gain new oncogenic functions, defined as gain-of-function (GOF), which enable them to promote tumorigenesis, metastasis and chemoresistance [11,12]. Therefore, we hypothesized that p53 mutation could be an ideal target to restore the sensitivity of HCC resistant cells to arsenic trioxide and inhibit HCC tumor metastasis.

Nutlin-3, a novel MDM2 inhibitor, has been shown to inhibit the p53-MDM2 or p73-MDM2 interaction, leading to the stabilization of p53 or p73 protein [10,13]. Furthermore, Nutlin-3 has also been reported to interfere with p-gp-mediated drug efflux for acting as a transporter substrate [14]. It revealed a potential therapeutic way for HCC resistant cells, especially in combination with arsenic trioxide. We designed this study to investigate the underlying mechanism of arsenic trioxide resistance and to evaluate whether Nutlin-3 could reverse the resistance.

## Results

### Effects of arsenic trioxide and Nutlin-3 on parental and arsenic trioxide resistant HCC cell lines

**Figure 1:**
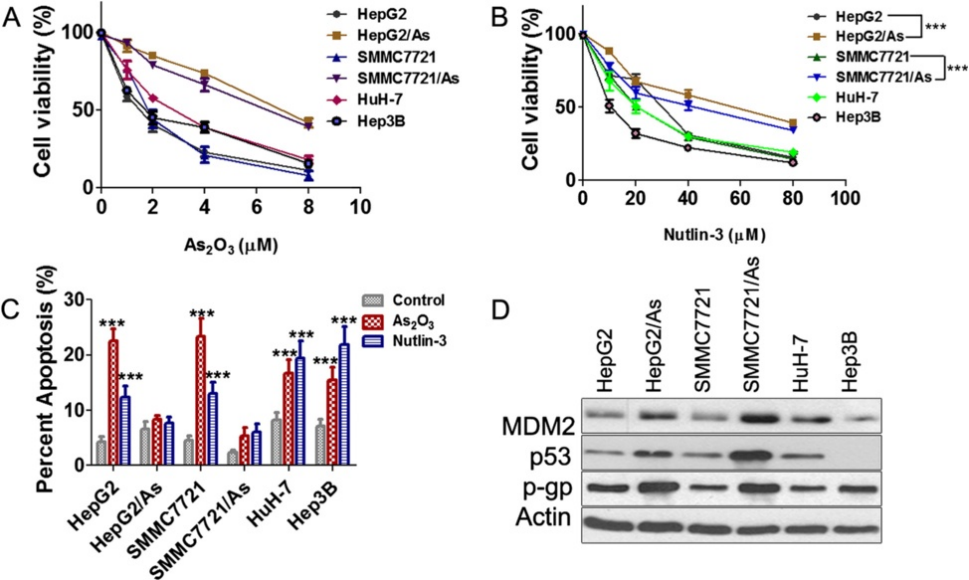
**Influence of arsenic trioxide or Nutlin-3 on HCC cell viability and apoptosis.** All HCC cell lines were treated with arsenic trioxide or Nutlin-3 for 48 h and 72 h respectively. **(A and B)** The cell viability was determined using MTT assay. Data are presented as mean ± SD, ***: *P* < 0.001. **(C)** The apoptosis was examined using Annexin V-FITC Apoptosis Detection Kit. ***: *P* < 0.001 compared with the control. **(D)** The expression of MDM2, p53 and p-gp was investigated by Western blot in indicated cell lines.

The sensitivity of HepG2, SMMC7721, HuH-7, Hep3B, HepG2/As and SMMC7721/As cells to arsenic trioxide or Nutlin-3 was examined by MTT assay after incubation with arsenic trioxide (48 h) or Nutlin-3 (72 h) respectively (Figure 1A,B). The IC_50_ of arsenic trioxide in HepG2/As or SMMC7721/As cells was 2.76 folds or 2.18 folds higher than that in the parental HepG2 or SMMC7721 cells respectively (Additional file 1: Table S1). The arsenic trioxide resistant cell lines, HepG2/As and SMMC7721/As, were insensitive to Nutlin-3; and the IC_50_ was 1.9 and 1.77 fold higher than that in HepG2 and SMMC7721 cells respectively (Additional file 1: Table S1). Arsenic trioxide (2 μM) induced significant apoptosis in HepG2 and SMMC7721 cells, but not in the SMMC7721/As or HepG2/As cells (Figure 1C). Nutlin-3 could also induce apoptosis in the parental HCC cells, but not in the resistant cells (Figure 1C). As in our previous study [5], the expression of MDM2, p-gp, and p53 were all increased in arsenic resistant cells compared with that in the parental cells (Figure 1D).

### Acquired mutation of p53 contributed to arsenic trioxide resistance in HCC

**Figure 2:**
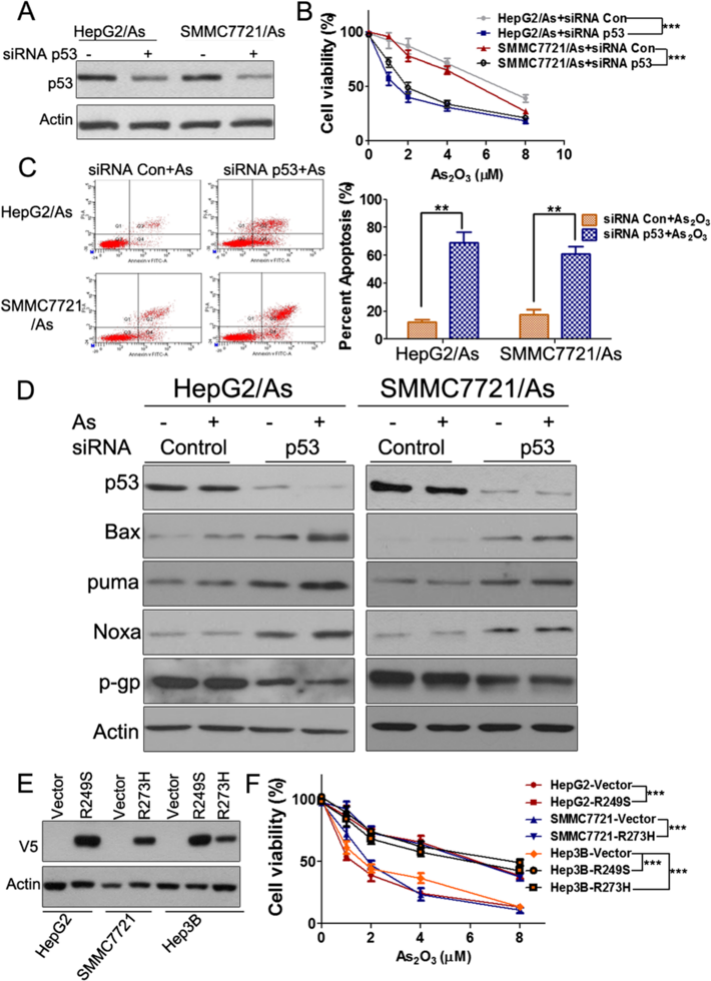
**Mutp53 plays an important role in HCC arsenic trioxide resistance. (A)** Knockdown of mutp53 in HCC resistant cells. **(B)** MTT assays were performed after cells were treated with arsenic trioxide for 48 h. ***: *P* < 0.001 versus negative siRNA-transfected cells. **(C)** Cells were treated with arsenic trioxide (2 μM) for 48 h before performing apoptosis assay. **, *P* < 0.01 versus negative siRNA-transfected cells. Histograms represent averages of three independent experiments. **(D)** Cells were treated with arsenic trioxide (2 μM) for 24 h after siRNA transfection (48 h). The target proteins were detected by Western blot analyses. **(E)** One representative Western blot from three independent experiments demonstrates the accumulation of the V5-tagged exogenous mutant p53 in each cell lines. **(F)** MTT assays were performed after cells were treated with arsenic trioxide for 48 h. ***: *P* < 0.001 versus vector control cell lines.

Then, we analyzed the mutation status of p53 gene in HepG2/As and SMMC7721/As cells. Results indicated that the two cell lines exhibited different mutations (Additional file 2: Table S2), which raised the question about the exact role of mutp53 in arsenic trioxide resistance. To address this question, we used siRNA to knock down mutp53 in HepG2/As and SMMC7721/As cells before these cells were exposed to arsenic trioxide treatment. Results indicated that siRNA significantly decreased the level of mutp53 in HepG2/As and SMMC7721/As cells (Figure 2A). The results of MTT assay suggested that p53 knockdown significantly increased the sensitivity of HepG2/As and SMMC7721/As cells to arsenic trioxide (Figure 2B). Apoptosis assays showed that p53 knockdown significantly increased the percentage of apoptotic cells after arsenic trioxide treatment (Figure 2C). Western blot results suggested that downstream genes of p73 (Noxa and puma) were increased after p53 knockdown (Figure 2D). Previous studies have suggested that mutp53 could upregulate multidrug resistance protein 1 (MDR1) gene expression [15]. Consistently, we found that the expression of p-gp was notably decreased by p53 knockdown (Figure 2D). To further determine the significance of mutp53 in arsenic trioxide resistance, we introduced Lenti-mutp53 plasmid with V5 tag into HepG2, SMMC7721 and Hep3B cells. The levels of exogenous V5-tagged mutant p53 protein were determined using Western blot after selection, as shown in Figure 2E. The MTT assays demonstrated that the overexpression of mutp53 obviously decreased the sensitivity of HCC cells to arsenic trioxide and resulted in arsenic trioxide resistance compared with the vector control (Figure 2F). Taken together, these results supported the hypothesis that p53 mutations were responsible for arsenic trioxide resistance in HCC.

### Mutp53 promotes migration and invasion in arsenic trioxide resistant HCC cells

**Figure 3:**
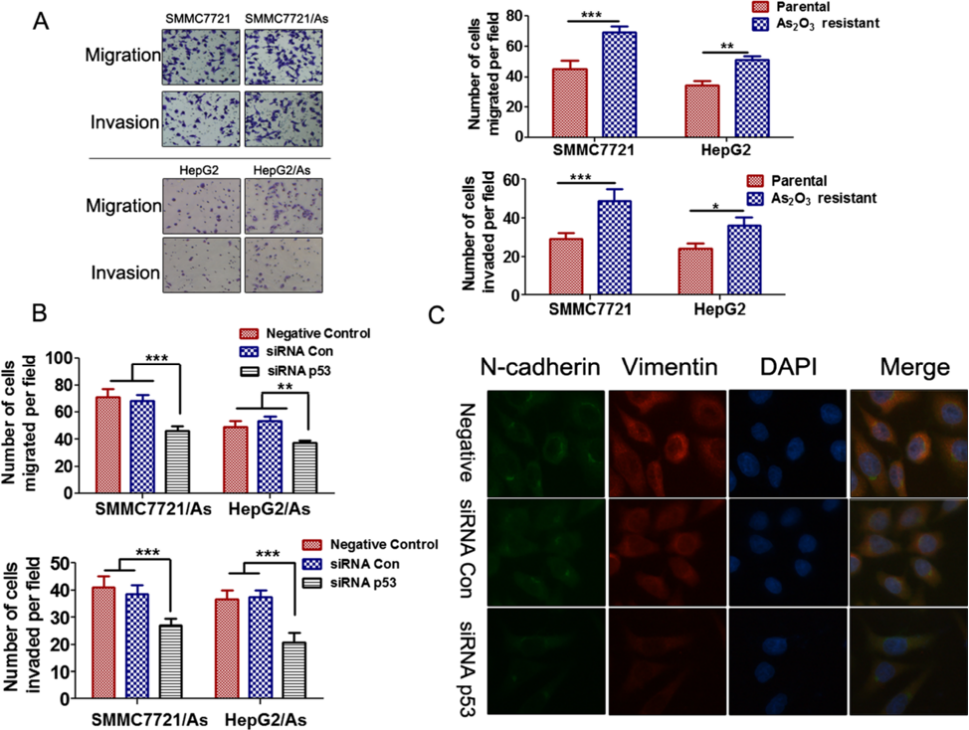
**Mutp53 contributed to the increased metastatic potential of HCC resistant cells. (A)** The migration and invasive abilities of HCC parental and arsenic trioxide resistant cells were determined by trans-well assays in chambers coated with matrigel (for invasion assays) or without matrigel (for migration assays). Left panels: representative images; right panels: quantifications of average number of cells/field. *P < 0.05, **P < 0.01, ***P < 0.001, two-way ANOVA with Bonferroni post-test. **(B)** Results of the migration and invasion assays for the HepG2/As and SMMC7721/As cells transfected with p53 siRNA or control siRNA. (**P < 0.01, ***P < 0.001, two-way ANOVA with Bonferroni post-test). **(C)** Single and merged images were taken to show immunofluorescence staining of N-cadherin (green) and vimentin (red) accompanied by the cell nucleus (blue) stained by DAPI.

We have also compared the metastatic abilities between the arsenic trioxide resistant cells and the parental cells. Results of two chamber transwell assays indicated that HepG2/As and SMMC7721/As cells had stronger abilities of migration and invasion than the corresponding parental cell lines (Figure 3A). Mutp53 has been illustrated to promote tumor metastasis in different cancers [11,12]. Consistent with previous reports, our results indicated that mutp53 knockdown dramatically decreased the migration and invasion abilities of arsenic trioxide resistant cells, suggesting that mutp53 plays an important role in the enhanced metastatic potential (Figure 3B). Given that mutp53 promotes migration and invasion in arsenic trioxide resistant HCC cells, we further investigated the effect of mutp53 on epithelial-mesenchymal transition (EMT), a critical event in tumor metastasis. Western blot results showed higher expression of E-cadherin, lower expression of N-cadherin and vimentin in HepG2/As and SMMC7721/As cells transfected with p53 siRNA compared with cells transfected with control siRNA (Additional file 3: Figure S1). Consistent with the results of immunofluorescence, p53 knockdown markedly reduced the levels of N-cadherin and vimentin in HepG2/As and SMMC7721/As cells (Figure 3C).

### Combination of arsenic trioxide and Nutlin-3 induces more apoptosis and synergistically inhibits the metastasis to lung

**Figure 4:**
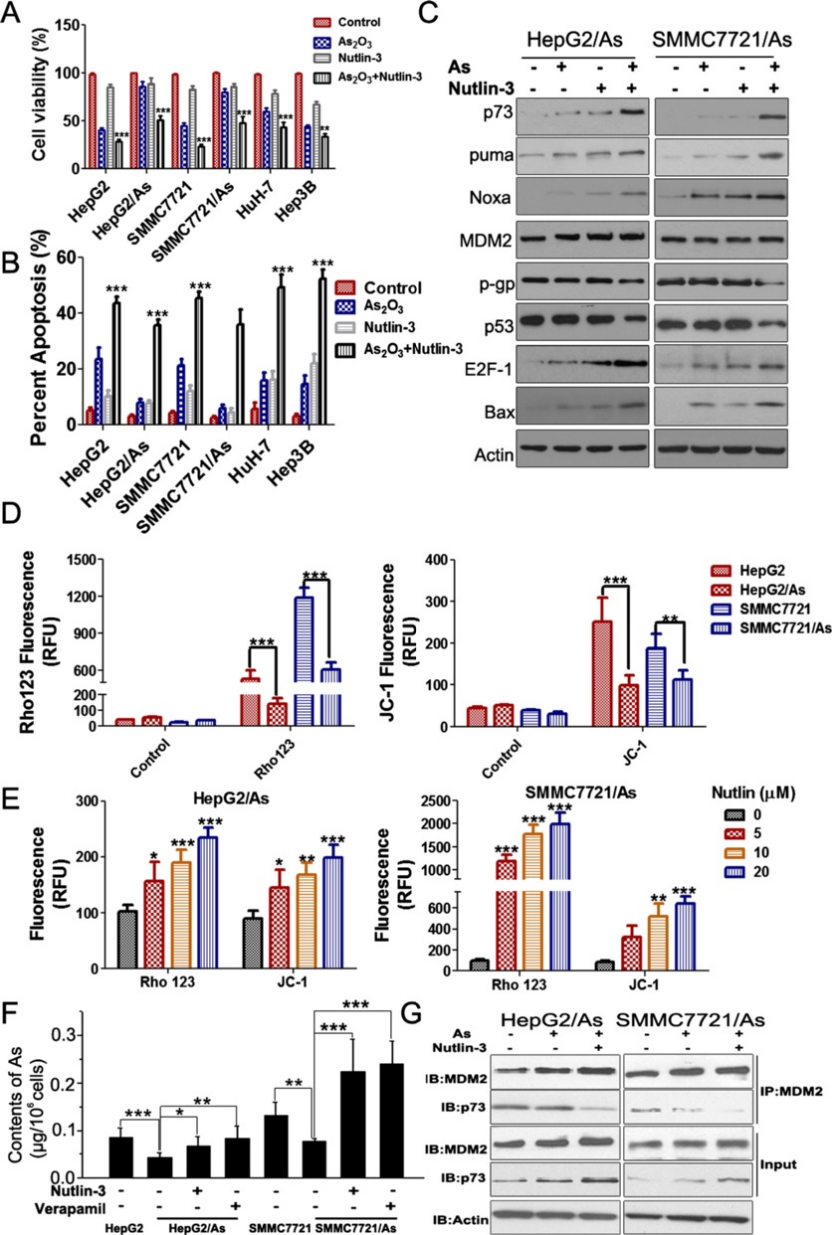
**The synergetic effects of arsenic trioxide and Nutlin-3 on HCC resistant cells. (A and B)** The concentration of arsenic trioxide applied here was 2 μM, and Nutlin-3 was 20 μM. Cell viability and apoptosis were determined after treatment for 48 h. **P < 0.01, ***P < 0.001, versus cells treated with arsenic trioxide or Nutlin-3 alone. **(C)** Protein expression was compared among untreated, arsenic trioxide, Nutlin-3 and Nutlin-3/arsenic trioxide groups in HepG2/As and SMMC7721/As cells. Cells had been cultured in the medium without any arsenic trioxide for at least one week. The concentration of rhodamine 123 and JC-1 was 0.1 μM. **(D)** The fluorescence of rhodamine 123 and JC-1 in HepG2/As and SMMC7721/As cells. Cells were incubated with rhodamine 123 and JC-1 for 60 min, and for another 60 min to efflux. **P < 0.01,***P < 0.001. **(E)** The inhibition of efflux was depended on the concentration of Nutlin-3. Nutlin-3 at different concentration was added with rhodamin 123 or JC-1 into medium and incubated for 60 min. Rho123, rhodamine 123. *P < 0.05, **P < 0.01, ***P < 0.001, versus control cells. **(F)** Nutlin-3 and R(+) verapamil assisted to increase the intracellular arsenic. All the cells were cultured with 2 μM arsenic trioxide, while treated groups were added 20 μM Nutlin-3 or 40 μM verapamil respectively and incubated for 2 h. *P < 0.05, **P < 0.01, ***P < 0.001. **(G)** Nutlin-3 inhibits binding of p73 to MDM2 when in combination with arsenic trioxide. Untreated cells, cells treated with 2μM arsenic trioxide, cells treated with 20 μM Nutlin-3, and cells treated with arsenic trioxide/Nutlin-3 combination for 24 h were immune-precipitated with an anti-MDM2 antibody. Immunocomplexes were subjected to immunoblotting with anti-MDM2 and anti-p73 antibodies.

Previous studies proved that Nutlin-3 showed synergistic effects when used in combination with other innovative drugs, such as TRAIL or bortozemib [16]. To investigate whether Nutlin-3 could cooperate with arsenic trioxide to inhibit tumor growth, we treated HCC cells with Nutlin-3, arsenic trioxide or both before MTT assays. The combination of Nutlin-3 (10 μM) and arsenic trioxide (2 μM) showed increased proliferation inhibition of HCC cells, approximate 41%-59% (Figure 4A). The apoptosis induction by Nutlin-3/arsenic trioxide combination was also significantly increased compared with that by each agent alone (Figure 4B). Western blots showed that although the expression of MDM2 had no apparent changes in the arsenic trioxide treated and combined groups (Figure 4C), the expression of E2F-1 and p73 was increased in HepG2/As and SMMC7721/As cells treated with the combination. The levels of p73 downstream genes, such as puma and Noxa were upregulated, and the Bcl-2 family member Bax, was also increased after arsenic trioxide treatment. Nutlin-3 can enhance the effects of arsenic trioxide on the above genes (Figure 4C). However, the decrease of mutp53 and p-gp could only be observed in the combined group. Accordingly, Nutlin-3 could cooperate with arsenic trioxide to inhibit the migration and invasion of arsenic trioxide resistant HCC cells (Additional file 4: Figure S2).

### Nutlin-3 could inhibit the function of p-gp and increase the intracellular arsenicals in resistant cells

The fluorescent p-gp substrates, rhodamine 123 and JC-1, were used to investigate the influence of Nutlin-3 on p-gp-mediated substance efflux as described [14]. Results indicated that rhodamine123 and JC-1 were pumped more from two arsenic resistant cells comparing to the corresponding parental cells (Figure 4D). We then examined whether Nutlin-3 could inhibit the efflux of fluorescent materials by p-gp. The results indicated that Nutlin-3 induced intra-cellular accumulation of rhodamine123 and JC-1 in HepG2/As and SMMC7721/As cells (Figure 4E). Subsequently, the gross of arsenical in cells was compared among HepG2, SMMC7721, HepG2/As and SMMC7721/As cell lines by atomic fluorescence spectrometry (Figure 4F). The intracellular arsenical in HepG2 was 0.0855 μg/10^6^ cells after cultured in 2 μM arsenic trioxide medium for 2 h, and was 2 fold higher than that in HepG2/As (0.042 μg/10^6^ cells). Similarly, the intracellular arsenical in SMMC7721 (0.131 μg/10^6^ cells) was 1.7 fold higher than that in SMMC7721/As (0.077 μg/10^6^ cells). However, the intracellular arsenical of HepG2/As was increased and reached 0.066 μg/10^6^ cells, when arsenic trioxide was applied with 20 μM Nutlin-3 for 2 h. The intracellular arsenical of SMMC7721/As was strongly accumulated and reached 0.224 μg/10^6^ cells after the treatment of both arsenic trioxide and Nutlin-3. It’s worth to note that 40 μM verapamil (a p-gp inhibitor) induced accumulation of arsenical in HepG2/As and SMMC7721/As (0.083 and 0.24 μg/10^6^ cells respective) as same as 20 μM Nutlin-3, which was coincident with our previous results [5].

### Nutlin-3 disrupts the binding of p73 with MDM2 when in combination with arsenic trioxide

As reported, p73 could bind to the MDM2 N-terminal hydrophobic pocket, which is the region targeted by Nutlin-3 [10]. Only the full length TAp73, but not DNp73 isoforms that lack the TA domain, would be expected to bind MDM2. Utilizing a TA-specific antibody raised against the N-terminus of p73, we performed co-immunoprecipitation to evaluate and confirm the effects of Nutlin-3 on the p73-MDM2 interaction in resistant cells. We found that in untreated, arsenic trioxide treated HCC resistant cells, p73 binds to MDM2. However, Nutlin-3 treatment largely inhibited this binding in the presence of arsenic trioxide (Figure 4G).

### Nutlin-3/arsenic trioxide combination could synergistically decrease mutant p53 in arsenic trioxide resistant HCC cells

The slight decrease of mutp53 by arsenic trioxide aforementioned in Figure 4C might provide a rationale for the new mechanism of mutp53 degradation mediated by Nutlin-3/arsenic trioxide combination. Recently, several studies reported that arsenic could degrade mutp53 or ΔNp63 [17,18]. In the current study, we treated the HepG2/As and SMMC7721/As cells with different doses of arsenic trioxide for 10 h. Results indicated that single use of arsenic trioxide could decrease mutp53 only if the concentration was above 8 μM (Additional file 5: Figure S3A), which was highly above the clinically relevant dose. To investigate whether Nutlin-3 could decrease the dose needed for mutp53 degradation by arsenic trioxide, resistant cells were treated with various doses of arsenic trioxide in the presence of Nutlin-3 (20 μM). The results showed that mutp53 was considerably decreased by arsenic trioxide at a dose as low as 2 μM (Additional file 5: Figure S3B). It might be due to the inhibition of p-gp by Nutlin-3, which enables more arsenic trioxide to enter the HCC resistant cells to degrade mutp53.

### Nutlin-3 potentiates the antitumor activity of arsenic trioxide in an orthotopic hepatic tumor model and cooperates with arsenic trioxide to inhibit the metastasis to lung

**Figure 5:**
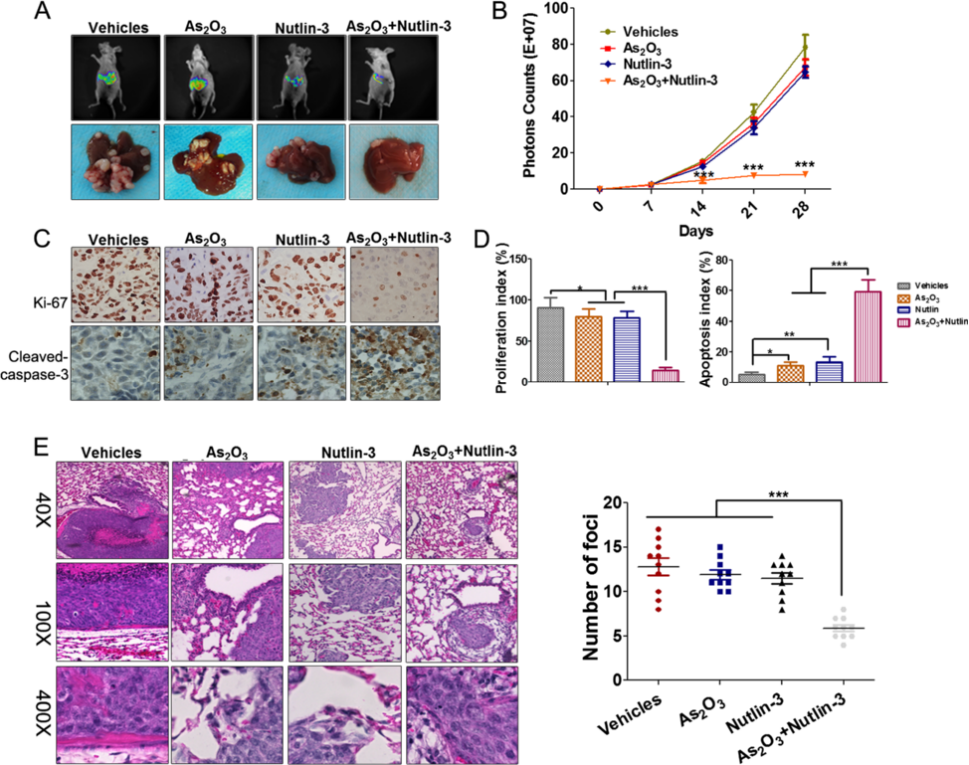
**Nutlin-3 potentiates the anti-tumor effects of arsenic trioxide *in vivo*. (A)** Representative bioluminescence images corresponding to the SMMC7721/As orthotopic hepatic tumors formed in the liver of the nude mice. **(B)** Volume of SMMC7721/As orthotopic tumors was determined at different time points. Data points represent the mean ± SD. (***P < 0.001, two-way ANOVA with Bonferroni post-test). **(C)** Representative images of sections stained with anti-Ki-67 antibody, or with anti-cleaved caspase-3 antibody. **(D)** Cells expressing Ki-67 were counted to calculate the proliferation index; cleaved caspase-3-positive cells were counted to give the apoptosis index. (*P < 0.05, **P < 0.01, ***P < 0.001, two-way ANOVA with Bonferroni post-test). **(E)** The representative H&E staining of pulmonary metastasis (arrows) on 5wk (left panel) and the average number of foci per mouse were calculated (right panel); (***P < 0.001, two-way ANOVA with Bonferroni post-test).

Based on the *in vitro* results, we designed experiments to determine the effects of Nutlin-3 and/or arsenic trioxide on orthotopically implanted hepatic tumors in nude mice. Luciferase-transfected SMMC7721/As cells were implanted in the liver of nude mice and the hepatic tumors were assessed by the bioluminescence IVIS on days 7, 14, 21, and 28. The bioluminescence imaging results (Figure 5A,B) indicated a gradual increase in tumor volume in the control group. Nutlin-3 or arsenic trioxide at the current dose could not efficiently inhibit the hepatic tumor growth. However, combination of Nutlin-3 and arsenic trioxide notably inhibited the tumor growth compared with the other three groups. We then examined the expression of the cell proliferation marker (Ki-67) and apoptosis marker (cleaved caspase-3) in tumor tissues. The results showed that combination of arsenic trioxide and Nutlin-3 significantly downregulated the expression of Ki-67 in tumor tissues compared with the groups treated with arsenic trioxide or Nutlin-3 alone (Figure 5C,D). The results also showed that only the combination could induce significant apoptosis as suggested by the cleaved caspase-3 staining (Figure 5C,D). In addition, the levels of p53 and p-gp in the tumor tissues had also been assessed using Western blot. As shown in Additional file 6: Figure S4, the results were consistent with that *in vitro,* which further strengthened the conclusions based on the *in vitro* data. To examine the therapeutic efficacy of arsenic trioxide and Nutlin-3 against tumor metastasis, HepG2/As cells were injected into nude mice via tail vein to imitate tumor lung metastasis. We found that when arsenic trioxide or Nutlin-3 was administrated alone, the average number of foci per mouse was not significantly reduced. However, the average number of foci was obviously reduced in the combination group (Figure 5E), suggesting arsenic trioxide and Nutlin-3 could synergistically inhibit the metastasis to lung.

## Discussion

Anti-cancer drug resistance is always a big problem obstructing the development of cancer chemotherapy. Usually, resistant cancer cells might have more than two bio-functions that help them to evade the lethal attack of chemotherapy. This is why we have not received any encouraging result from the use of competitive inhibitor of drug efflux. In the current study, we reported that a series of stepwise arsenic trioxide selections could induce p53 mutations in HCC cells, which led to the activation and stabilization of mutp53. The results further suggested that mutp53 might take part in the acquired arsenic resistance and the increased metastatic abilities of resistant HCC cells. It has been reported previously that p73 could be induced by a wide variety of chemotherapeutic drugs, including arsenic trioxide [9]. P73, which shares sequence homology with p53, is rarely mutated in human cancer, but has been reported to be inactivated by mutp53 or MDM2 [19,20]. We found the same phenomenon that p73 could be induced by arsenic trioxide in HCC cells here. To reactivate p73, two possible ways, Nutlin-3 or mutp53 knockdown, alone or in combination with arsenic trioxide were applied in the treatment of resistant HCC cells. Nutlin-3, a small-molecule MDM2 inhibitor, has been reported as a novel p53 activator in many malignant tumors [16]. Our results indicated that Nutlin-3 was effective in primitive HCC cells, while arsenic resistant cells showed resistance to it. Interestingly, either Nutlin-3 or p53 knockdown combined with arsenic trioxide could induce significant growth inhibition and apoptosis in arsenic trioxide sensitive and resistant HCC cells. We further found that the synergistic effects were due to the activation of p73 pathway and the competitive inhibition of drug efflux.

Recently, several studies reported that Nutlin-3, used alone or in combination with other chemotherapeutic drugs, such as sorafenib or Dasatinib could promote the synergistic cytotoxicity, irrespectively of p53 status [21,22]. Zauli *et al.* demonstrated that Nutlin-3 plus Dasatinib showed significant synergistic antileukemic effects in both p53 wild-type and p53 mutated B chronic lymphocytic leukemias. These studies suggested that the combination of Nutlin-3 and other innovative drugs might offer a novel therapeutic strategy for tumors with p53 mutation, which usually have a worse prognosis [22]. Moreover, Nutlin-3 was also found to be a substrate of p-gp and inhibit the drug efflux of p-gp [14]. We proved that Nutlin-3 could inhibit the efflux of rhodamine 123 and JC-1 by p-gp in a concentration dependent manner. Furthermore, Nutlin-3 could also inhibited the efflux of arsenic and gathered intracellular arsenic in HepG2/As and SMMC7721/As cells. Nutlin-3 could not induce significant growth inhibition in arsenic resistant cells, despite non-resistant cells were sensitive to it. The over efflux of Nutlin-3 from resistant cells by p-gp might be the reason of this insensitivity. As same as arsenical, it is difficult to reach the threshold of lethal dose for intracellular Nutlin-3 in arsenic resistant cells under a low concentration of Nutlin-3. However, the combination of Nutlin-3 and arsenic trioxide effectively overcome the drug resistance through both competitive inhibition of drug efflux and interference of p73-MDM2 interaction. Taken together, these results suggested that the combination of Nutlin-3 and cytotoxic agent would be a promising way to treat malignant cells, even resistant ones.

According to the results of the current study, targeting either p-gp or negative p73 regulators could be a method to solve drug resistance in p53 mutant HCC cells, but it is not the most effective one. As resistant cancer cells have multiple ways to evade cell death, we should find a manner to block those ways simultaneously. In this study, besides the finding that Nutlin-3 could inhibit the drug efflux and the activity of MDM2, we found that arsenic trioxide could also degrade mutp53, but only at a high dose. Nutlin-3 could decrease the dose of arsenic trioxide needed for mutp53 degradation, possibly through the inhibition of p-gp. It is possible that solid tumor with mutp53 may have the same mechanisms for drug resistance. This suggests that Nutlin-3 could assist arsenic trioxide or other anticancer drugs to treat malignant disease, especially when drug resistance appears. The *in vivo* experiments indicated that the administration of Nutlin-3 or arsenic trioxide alone could not inhibit the growth or metastasis of arsenic trioxide resistant tumors in nude mice models. However, when the two agents were used in combination, the tumor growth and metastasis were significantly inhibited and almost completely eliminated *in vivo*.

## Conclusions

**Figure 6:**
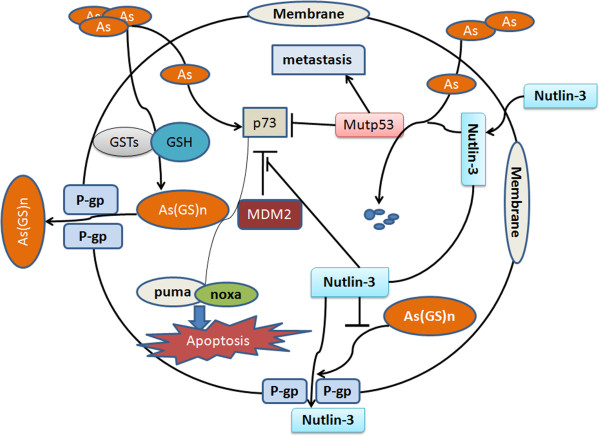
A schematic presentation for the possible mechanisms of arsenic trioxide resistance in HCC and the synergistical anticancer mechanisms of arsenic trioxide/Nutlin-3 combination in the current study.

Herein, the major findings of the present study are: a) acquisition of p53 mutation contributed to the resistance of HCC cells to arsenic trioxide; b) activation of p73 by p53 knock down or Nutlin-3 could overcome arsenic trioxide resistance; c) Nutlin-3/arsenic trioxide combination could inhibit the tumor growth and metastasis of arsenic trioxide resistant HCC cells effectively through mutp53 degradation and activating p73 both *in vivo* and *in vitro* (Figure 6). In conclusion, this study suggests that the combined treatment of arsenic trioxide plus Nutlin-3 might offer a novel therapeutic approach for HCC, especially arsenic trioxide resistant ones that have bad prognosis and urgently need innovative therapeutic strategies.

## Materials and methods

### Reagents

Arsenic trioxide (Sigma, USA) was stocked at 2 mM and stored at 4°C. Nutlin-3 (Cayman, USA) was dissolved in DMSO at 40 mM and stored at -20°C. R (+) Verapamil (Sigma, USA) was stocked at 1 mM and stored at 4°C. Rhodamin 123, JC-1 and DAPI were purchased from Beyotime (Shanghai, China). RPMI1640, DMEM and fetal bovine serum were also purchased from Invitrogen.

### Cell culture

The p53 status of HepG2, HuH-7 and Hep3B cell lines was confirmed in IARC TP53 Database ( http://p53.iarc.fr/CellLines.aspx) or reference [23]. HepG2/As and SMMC7721/As cell lines were isolated from HepG2 and SMMC7721 cell lines, by a series of stepwise selections via treatment with increasing concentrations of arsenic trioxide as described previously [5]. HepG2, HepG2/As, SMMC7721, and SMMC7721/As were cultured in DMEM with 10% FBS. HepG2/As and SMMC7721/As were maintained in medium containing 2 μM arsenic trioxide. HuH-7 cell line was purchased from Xiangya central experiment laboratory (Zhongnan University, China), and Hep3B cell line was purchased from American Type Culture Collection (ATCC). The firefly luciferase gene plasmid-pGL3 vector (Promega, Madison, WI) was transfected into SMMC7721/As cells to establish the SMMC7721/As-Luci stable cell line after selection.

### Cytotoxicity assay

Cytotoxicity assays were performed using MTT assay as described previously [5,24].

### Western blotting

Standard Western blot assays were performed as described [5,24]. The following antibodies were used for immunoblotting: anti-β-actin (C4), anti-p-gp (D-11), anti-p53 (FL-393), anti-Bax, anti-p73 (H-79), anti-E2F-1 (KH95) were all purchased from Santa Cruz Biotechnology. The MDM2 antibody (2A10), N-cadherin, E-cadherin and vimentin, PUMA and Noxa antibodies were all purchased from Abcam.

### Flow cytometry

To investigate ABC transporter–mediated substance efflux, cells were incubated with rhodamine 123 (0.1 μmol/L) and JC-1 (0.1 μmol/L) for 60 min. Then, cells were washed with PBS and incubated for another 60 min before analyzed by flow cytometry (FACSCalibur, BD) as described previously [14]. Similarly, to examine the function of Nutlin-3, 5-20 μM Nutlin-3 was applied simultaneously with rhodamine 123 and JC-1 for 60 min. Fluorescence was analyzed after washing and 60 min incubation. To analyze the apoptosis induced by arsenic trioxide or Nutlin-3, Annexin V-FITC Apoptosis Detection Kit (BD Biosciences) was used according to the protocol. The cell cycle distribution was determined using the CycleTEST™ PLUS DNA Reagent Kit (BD Biosciences) according to the protocol and analyzed by flow cytometry (Beckman Coulter FC500).

### SMMC7721/As orthotopic hepatic tumor experiments

SMMC7721/As-luciferase-transfected (Luci-SMMC7721/As) cells were harvested from subconfluent cultures and washed once in serum-free medium and resuspended in PBS. Only suspensions consisting of single cells, with > 90% viability, were used for the injections. Luci-SMMC7721/As cells (5 × 10^6^) were implanted into the liver of the nude mice as described [24]. After one week of implantation, mice were randomized into the following treatment groups (n = 10/group) based on the bioluminescence measured after IVIS imaging: (a) Control (treated with vehicles); (b) arsenic trioxide (i.p., 5 mg/kg/d); (c) Nutlin-3 (p.o., 200 mg/kg, twice a day); (d) arsenic trioxide (i.p., 5 mg/kg/d) + Nutlin-3 (p.o., 200 mg/kg, twice a day). Mice were imaged by the bioluminescence IVIS Imaging System weekly and then mice were sacrificed.

#### *In vivo* metastasis analysis

HepG2/As cells (1 × 10^6^/0.2 ml) were injected into nude mice by way of tail vein to imitate tumor metastasis. Experimental animals were randomized into the following treatment groups (n = 10/group): (a) Control (treated with vehicles); (b) arsenic trioxide (i.p., 5 mg/kg/d); (c) Nutlin-3 (p.o., 200 mg/kg, twice a day); (d) arsenic trioxide (i.p., 5 mg/kg/d) + Nutlin-3 (p.o., 200 mg/kg, twice a day). The mice were killed 5 weeks after the inoculation and lungs were removed and fixed in formaldehyde. The lung metastases were confirmed by H&E staining.

#### Statistical analysis

Statistical analyses were performed with the GraphPad Prism software package (v. 4.02; GraphPad Prism Software Inc, San Diego, CA) or SPSS 16.0 software (SPSS, Chicago, IL, USA). Values were expressed as mean ± SD values using the Student’s *t*-test and one-way ANOVA with the Bonferroni’s correction when comparing more than two groups. *P* < 0.05 was considered to be statistically significant.

Detailed description of other Materials, Methods and the primers used for p53 mutation detection can be found in the Additional file 7 and Additional file 8: Table S3.

## Abbreviations

HCC: Hepatocellular carcinoma; ABC: ATP-binding Cassette; MTT: 3-(4,5-dimethylthiazol-2-yl)-2,5-diphenyltetrazolium bromide; MDR1: Multidrug resistance protein 1; p-gp: p-glycoprotein.

## Competing interest

The authors have declared that no competing interests exist.

## Authors’ contributions

ZYL, DLY and TSZ carried out the majority of the experiments in this study. LXL, HCJ and TSZ designed study, and wrote manuscript. JBW, YJL, XC, YJL, XS and SYQ carried out protein interaction and siRNA experiments and edited manuscript. RPS, BSS and HYY carried out nude mice experiments. XZM, SHP, JRL carried out the IF and IHC experiments. JRL participated in study design and gave critical discussions. TSZ edited manuscript. All authors read and approved the final manuscript.

## Supplementary Material

Additional file 1: Table S1 IC_50_ of arsenic trioxide or Nutlin-3 in different HCC cell lines. IC_50_ was examined using MTT assay. HCC cells were treated with arsenic trioxide for 48 h or Nutlin-3 for 72 h. Values are means ± SD of at least three independent experiments performed in triplicate. Click here for file

Additional file 2: Table S2 p53 mutations in arsenic trioxide resistant HCC cell lines. Click here for file

Additional file 3: Figure S1 The levels of E-cadherin, N-cadherin and Vimentin in the SMMC7721/As and HepG2/As cells without treatment or cells transfected with p53 siRNA or control siRNA was examined by western blotting assays. Click here for file

Additional file 4: Figure S2 The results of migration and invasion assays in HepG2/As or SMMC7721/As cells after arsenic trioxide, Nutlin-3 or arsenic trioxide/Nutlin-3 treatment for 24 h. (*P < 0.05, ***P < 0.001, two-way ANOVA with Bonferroni post-test). Click here for file

Additional file 5: Figure S3 Nutlin-3 cooperates with arsenic trioxide to degrade mutp53 protein in HCC arsenic trioxide resistant cells. (A) Western blots were prepared with extracts from HCC resistant cells untreated or treated with different concentrations of arsenic trioxide for 10 h, and then probed with antibodies against p53 and actin. (B) Western blots were prepared with extracts from HCC resistant cells untreated or treated with arsenic trioxide of different concentrations in the presence of Nutlin-3 for 10 h, and then probed with antibodies against p53 and actin. Click here for file

Additional file 6: Figure S4 The expression of p53 and p-gp in the orthotopic hepatic tumor tissues from the indicated group was examined by western blot. Actin was used as internal control. Click here for file

Additional file 7 Supplemental materials, methods and figure legends. Click here for file

Additional file 8: Table S3 The sequences of the primers used for p53 mutation analysis. Click here for file
