# Supplementary figures and images for: Nutlin-3 overcomes arsenic trioxide resistance and tumor metastasis mediated by mutant p53 in Hepatocellular Carcinoma
Source: Mol Cancer. 2014 May 31;13:133. doi: 10.1186/1476-4598-13-133 (PMC4046148; doi:10.1186/1476-4598-13-133)

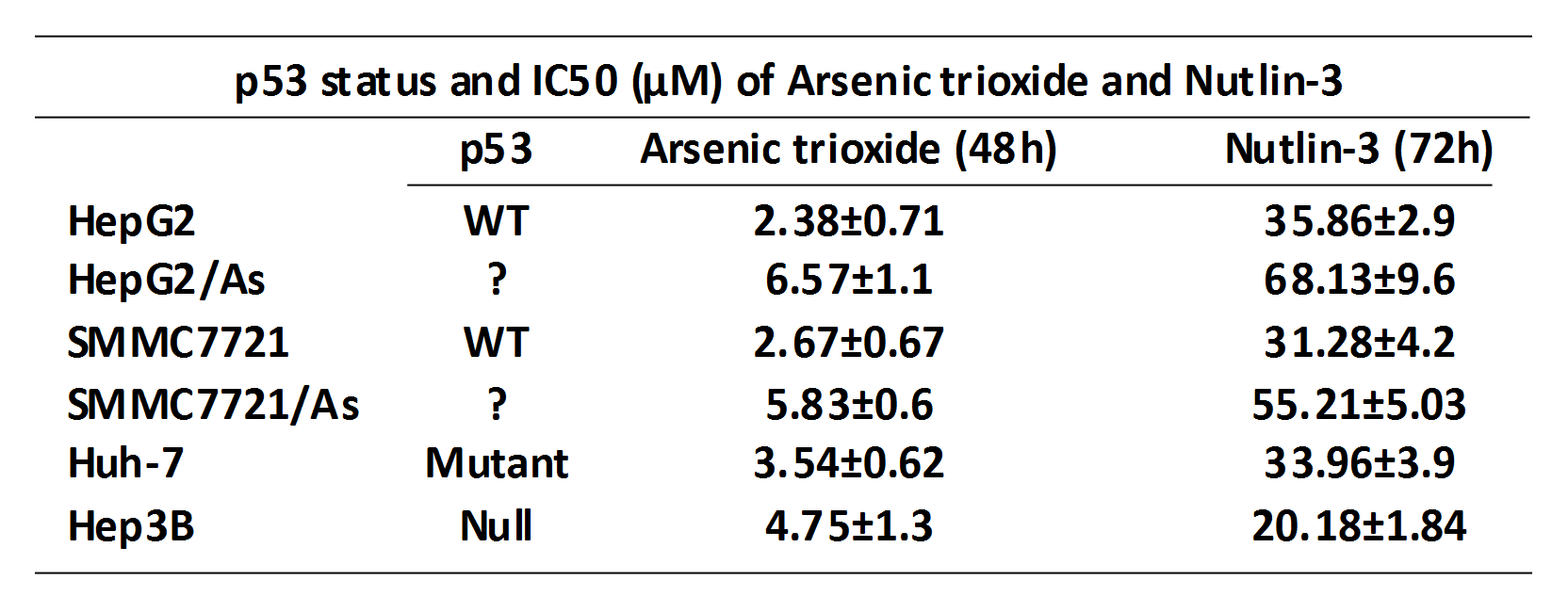

Supplement: Additional file 1: Table S1 — IC50 of arsenic trioxide or Nutlin-3 in different HCC cell lines. IC50 was examined using MTT assay. HCC cells were treated with arsenic trioxide for 48 h or Nutlin-3 for 72 h. Values are means ± SD of at least three independent experiments performed in triplicate. [file 1476-4598-13-133-S1.tiff]

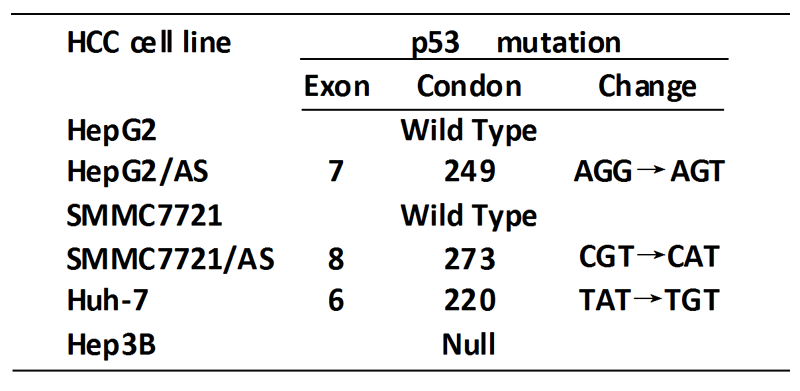

Supplement: Additional file 2: Table S2 — p53 mutations in arsenic trioxide resistant HCC cell lines. [file 1476-4598-13-133-S2.tiff]

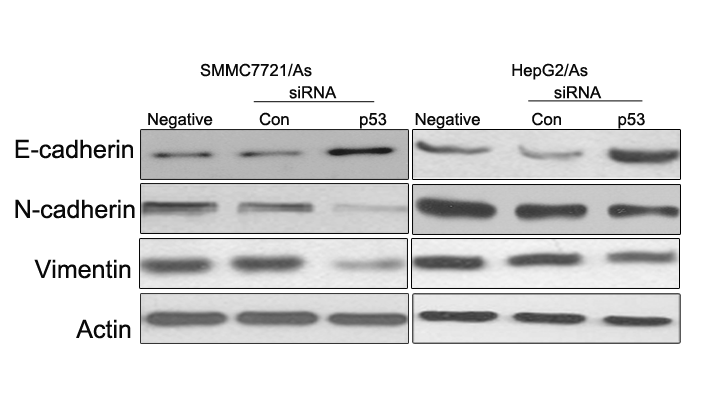

Supplement: Additional file 3: Figure S1 — The levels of E-cadherin, N-cadherin and Vimentin in the SMMC7721/As and HepG2/As cells without treatment or cells transfected with p53 siRNA or control siRNA was examined by western blotting assays. [file 1476-4598-13-133-S3.tiff]

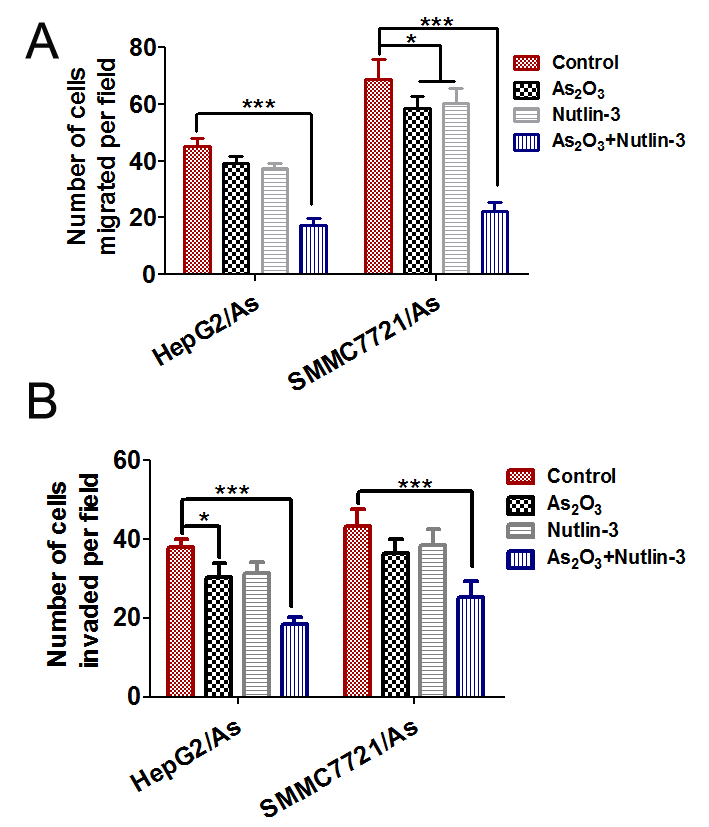

Supplement: Additional file 4: Figure S2 — The results of migration and invasion assays in HepG2/As or SMMC7721/As cells after arsenic trioxide, Nutlin-3 or arsenic trioxide/Nutlin-3 treatment for 24 h. (*P < 0.05, ***P < 0.001, two-way ANOVA with Bonferroni post-test). [file 1476-4598-13-133-S4.tiff]

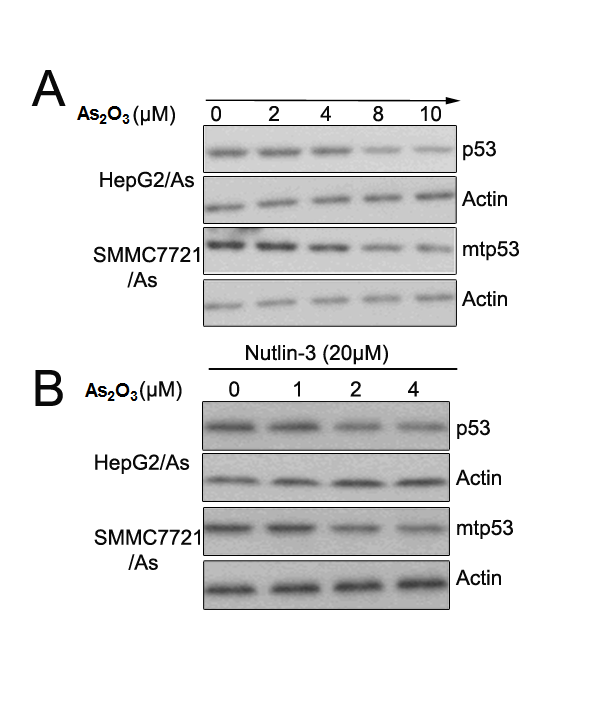

Supplement: Additional file 5: Figure S3 — Nutlin-3 cooperates with arsenic trioxide to degrade mutp53 protein in HCC arsenic trioxide resistant cells. (A) Western blots were prepared with extracts from HCC resistant cells untreated or treated with different concentrations of arsenic trioxide for 10 h, and then probed with antibodies against p53 and actin. (B) Western blots were prepared with extracts from HCC resistant cells untreated or treated with arsenic trioxide of different concentrations in the presence of Nutlin-3 for 10 h, and then probed with antibodies against p53 and actin. [file 1476-4598-13-133-S5.tiff]

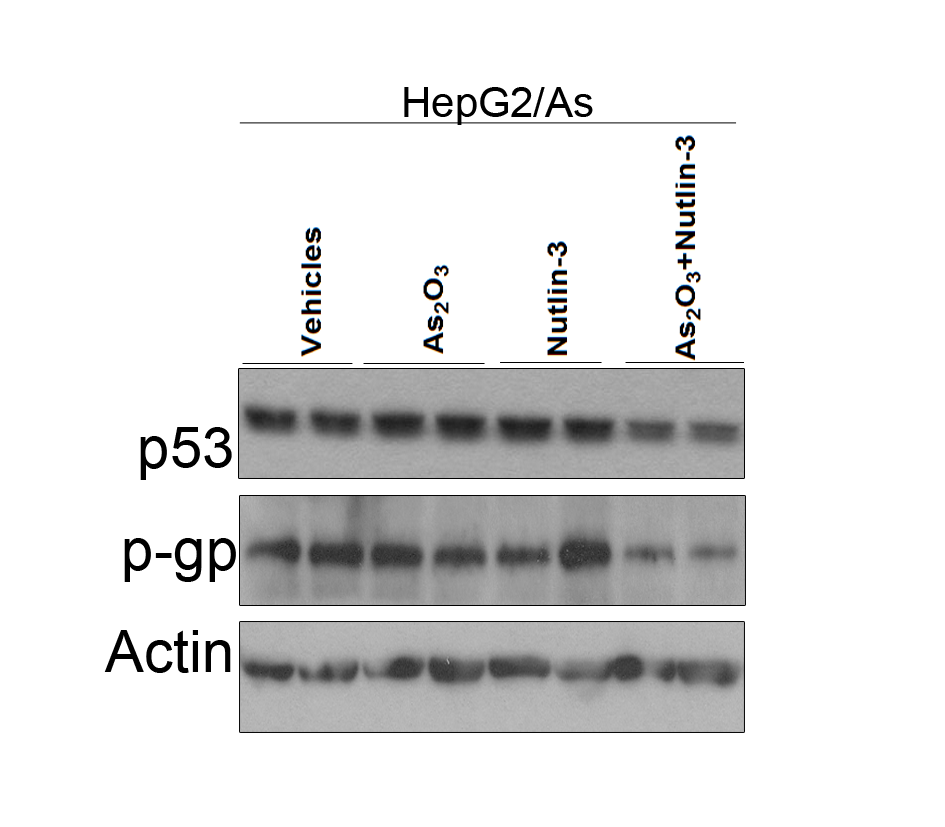

Supplement: Additional file 6: Figure S4 — The expression of p53 and p-gp in the orthotopic hepatic tumor tissues from the indicated group was examined by western blot. Actin was used as internal control. [file 1476-4598-13-133-S6.tiff]

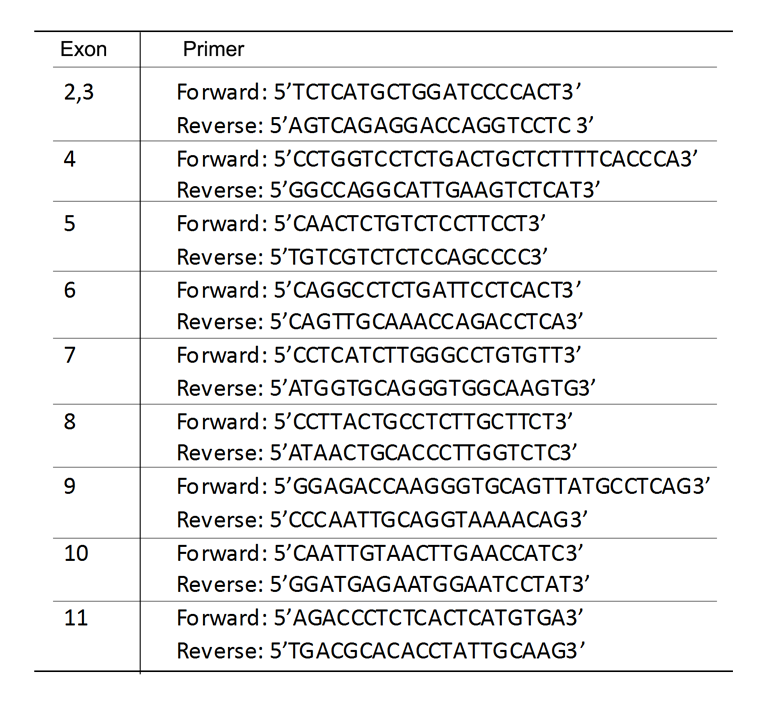

Supplement: Additional file 8: Table S3 — The sequences of the primers used for p53 mutation analysis. [file 1476-4598-13-133-S8.tiff]
