# Supplementary material for: Nutlin-3 overcomes arsenic trioxide resistance and tumor metastasis mediated by mutant p53 in Hepatocellular Carcinoma
Source: Mol Cancer. 2014 May 31;13:133. doi: 10.1186/1476-4598-13-133 (PMC4046148; doi:10.1186/1476-4598-13-133)
Supplement: Additional file 7 — Supplemental materials, methods and figure legends. [file 1476-4598-13-133-S7.doc]

**Nutlin-3 overcomes arsenic trioxide resistance and tumor metastasis mediated by mutant p53 in Hepatocellular Carcinoma**

**Authors:**

Tongsen Zheng*^1^, Dalong Yin*^1^, Zhaoyang Lu**†***^1^, Jiabei Wang*^1^, Yuejin Li^1^, Xi Chen^1^, Yingjian Liang^1^, Xuan Song^1^, Shuyi Qi^2^, Boshi Sun^1^, Changmin Xie^1^, Xianzhi Meng^1^, Shangha Pan^1^, Jiaren Liu^3^, Hongchi Jiang^1^, Lianxin Liu**†**^1^

*These authors contributed equally to this work.

**Affiliation For Authors:** ^1^Key Laboratory of Hepatosplenic Surgery, Ministry of Education, Department of General Surgery, the First Affiliated Hospital of Harbin Medical University, Harbin, China. ^2^Department of Gerontology, the First Affiliated Hospital of Harbin Medical University, Harbin, China. ^3^Children’s Hospital Boston and Harvard Medical School, Boston, 300 Longwood Ave, Boston, MA 02115–5737, USA .

**†Corresponding author:**

Lianxin Liu, M.D., Ph.D, Key Laboratory of Hepatosplenic Surgery, Ministry of Education, Department of General Surgery, the First Affiliated Hospital of Harbin Medical University, Harbin, China.

**Address:** #23 Youzheng Street, Harbin, 150001, Heilongjiang Province, China.

**Phone number :** +86-451-85553886

**Fax :** +86-451-53670428

**E-mail:** [liulianxin@ems.hrbmu.edu.cn](mailto:liulianxin@ems.hrbmu.edu.cn)

**OR**

Zhaoyang Lu, M.D., Ph.D, Key Laboratory of Hepatosplenic Surgery, Ministry of Education, Department of General Surgery, the First Affiliated Hospital of Harbin Medical University, Harbin, China.

**Address:** #23 Youzheng Street, Harbin, 150001, Heilongjiang Province, China.

**Phone number/Fax :** +86-451-53643628

**E-mail:** luzhaoyang112@yeah.net

**Email addresses for all authors:**

Tongsen Zheng (zhengtongsen@hotmail.com);

Dalong Yin (dalong48@163.com);

Zhaoyang Lu (luzhaoyang112@yeah.net);

Jiabei Wang (inn_2001@126.com);

Yuejin Li (liyuejin0203@126.com);

Xi Chen (fangxiang221@yeah.net);

Yingjian Liang (genomeliang@sina.com);

Xuan Song (tiemin2008@163.com);

Shuyi Qi (qishuyi118@126.com);

Boshi Sun (kobe007@qq.com);

Changming Xie (xiaxiaoxiaoming@126.com);

Xianzhi Meng (mengfeiyang01@hotmail.com);

Shangha Pan (panshanghazhongxin@126.com);

Jiaren Liu (jia-ren.liu@childrens.harvard.edu);

Hongchi Jiang (san3mudeepwhite@163.com);

Lianxin Liu (liulianxin@ems.hrbmu.edu.cn)

**Supplemental Data:**

**Supplemental Experimental Procedures:**

**p53 Mutation detection in HCC resistant cells**

The mutation detection of p53 was carried out by amplification of exons from genomic DNA with primers (Table S1) within flanking intron sequences and then direct sequencing of these fragments from both sides. These primers were used for both amplification and sequencing reaction. Amplication was performed with TaKaRa Ex T*aq* DNA polymerase (TaKaRa Shuzo, Kyoto). The amplified fragments were purified by polyethyleneglycol precipitation, then subjected to DNA sequencing in ABI PRISM 310 Genetic Analyzer (Applied Biosystems, Foster City, CA). If mutation were detected, DNA from the resistant cancer cells was extracted again, and amplified by PCR, followed by sequencing in both directions to confirm the mutations.

**Lentiviral particle and stable cell line generation**

The constracts of mutant p53 ([pLenti6/V5-p53-R273H](http://www.addgene.org/22934/) and [pLenti6/V5-p53-R249S](http://www.addgene.org/22935/)) were all purchased from Addgene plasmid repository (USA). Each construct was validated for the correct p53 mutation by DNA sequencing. Lentiviral particles were generated by individually transfecting 293FT cells with the two mutp53 pLenti6/V5 constructs and the ViraPower Packaging Mix with Lipofectamine 2000 according to the manufacturer’s protocol (Invitrogen). Stable mutant p53-expressing HCC cells were generated by infection with lentiviral particles at a 1:150 dilution in the growth media with 8μg/ml polybrene (Sigma-Aldrich). Selection of stably expressing mutp53 cell lines was conducted with 2 μg/ml blasticidin (Invitrogen).

**Immunofluorescence**

Briefly, cells seeded on coverslips were fixed with 4% (w/v) paraformaldehyde (Sigma) for 10 min and permeabilized with 0.1% (v/v) Triton X-100 for 5 min at room temperature. The cells were then incubated overnight with primary antibodies at 4°C, followed by incubation with fluorescent secondary antibody (invitrogen) for 1 hour at room temperature. After final washes with PBS, the coverslips were mounted using an anti-fade mounting solution containing 4',6-diamidino-2-phenylindole (DAPI; Vector lab, Burlingame, CA) and images were examined and captured.

**siRNA silence**

Control siRNA (sc-37007) and siRNA against p53 (sc-29435) was purchased from Santa Cruz Biotechnology. Cells were cultured in 6-well plates at a concentration of 60%-70% per well 24h before transfection. siRNA were transfected through Lipofectamine^TM^ 2000 as protocol (Invitrogen). After transfection, cells were cultured for 24h before subsequent examination or treatment.

**Cell migration and invasion assays**

Cell migration assays were performed in the BD Falcon 24-multiwell insert system (BD Biosciences, San Jose, CA, USA) according to the manufacturer's instruction. For the matrigel invasion assay, filters were precoated with 30μl Matrigel (BD Biosciences, USA) for 3h. Culture medium containing 10% FBS was added to lower chambers and aliquots of 3-7 × 10^4^ cells in 300μl of FBS-free medium were seeded into upper chambers. After the assays had been run for 24 h at 37 °C, non-migrated or non-invaded cells were removed from the upper surface of the filter. Cells on the lower surface of the membrane were fixed with ice-cold methanol and stained with crystal violet. Cell numbers were counted under an optical microscope. Each experiment was repeated at least three times.

**Intracellular arsenic assays**

Briefly, cells were treated with 2μM arsenic trioxide or combined with 20μM Nutlin-3 or 40μM R (+) Verapamil for 2h. Then cells were washed with cold PBS for 3 times, collected in 1.5ml tubes at a concentration of 1-2 x 10^6^ cells/ml, and frozen in -80˚C. Samples (0.5 ml) were mixed with 1.0ml perchloric acid and 4.0ml nitric acid, and heated to fuse. After cooled down, 1.0ml nitric acid and 1.0ml mixed solution (2% antiscorbic acid and 2% sulfourea) were added into the solution. The sample was analyzed by atomic fluorescence spectrometer (AFS-930, Ji Tian instrument Co., Ltd. Beijing).

**Co-Immunoprecipitation**

Cells were cultured to subconfluence in 10cm plates and treated with indicated drugs for 24h prior to harvesting. Cells were lysed in lysis buffer composed of 150mM NaCl, 50mM Tris (pH 7.4), 5mM EDTA, and 0.5% TritonX-100 supplemented with Complete Mini protease inhibitor. For immunoprecipitation, 500μg of protein was combined with 1μg of appropriate antibody and 10μl of Ultra-link immobilized A/G beads slurry (Pierce) and incubated overnight with rotation at 4°C.
